# Adolescent lipoprotein classifications according to National Health and Nutrition Examination Survey (NHANES) vs. National Cholesterol Education Program (NCEP) for predicting abnormal lipid levels in adulthood in a Middle East population

**DOI:** 10.1186/1476-511X-11-107

**Published:** 2012-08-31

**Authors:** Masumeh Hatami, Maryam Tohidi, Reza Mohebi, Davood Khalili, Fereidoun Azizi, Farzad Hadaegh

**Affiliations:** 1 Prevention of Metabolic Disorders Research Center, Research Institute for Endocrine Sciences, Shahid Beheshti University of Medical Sciences, Tehran, Iran; 2 Endocrine Research Center, Research Institute for Endocrine Sciences, Shahid Beheshti University of Medical Sciences, Tehran, Iran; 3 Anatomical and Clinical Pathology, Prevention of Metabolic Disorders Research Center, Research Institute for Endocrine Sciences (RIES), Shahid Beheshti University of Medical Science, P.O. Box: 19395–4763, Tehran, Islamic Republic of Iran

## Abstract

**Background:**

To compare the predictive ability of adolescent lipoprotein classification using the National Examination Survey (NHANES) cut points and those of the National Cholesterol Education Program (NCEP) for predicting abnormal levels in adulthood.

**Method:**

From 1032 adolescents, aged 14–19 years, participants of the Tehran Lipid and Glucose Study, all lipid measures were determined at baseline and again after 6 years. Multivariable Odds Ratios (ORs) were calculated for borderline and high categories of lipids to predict dyslipidemia in adulthood, considering the normal level as a reference. Area under the receiving characteristics curve (AUC) was used to assess the predictive ability of each adolescent lipid classification.

**Result:**

Applying the NCEP classification, the prevalences of high total cholesterol (TC), low-density lipoprotein cholesterol (LDL-C), triglycerides and low high density lipoprotein cholesterol (HDL-C) in males were 12.1%, 12.9%, 26.1% and 34.2% respectively; in females the corresponding prevalences were 15.4%, 17.9%, 21.4% and 25.0%, respectively. Using NHANES cut points, the prevalence of high TC, LDL-C and triglycerides were lower, than those defined by NCEP; the ORs of high categories of lipids (defined by NHANES) were higher than ORs based on the NECP classification, except for HDL-C. For all lipid measures, both classifications had similar predictive abilities, except for TC/HDL-C, which had higher predictive power applying the NHANES classification rather than the NCEP one (AUC 71% vs. 68%, respectively).

**Conclusion:**

No differences were found between NCEP and NHANES classifications for prediction of adult dyslipidemia, except for TC/HDL-C. Because of their simple application, NCEP cut points can be used in clinical settings.

## Introduction

Dyslipidemia is a major risk factor for cardiovascular disease (CVD) in adults worldwide [1,2]. Several studies showed that high levels of lipids during childhood are also associated with subclinical atherosclerosis and hyperlipidemia in adulthood [3]; almost 40-55% of children with abnormal lipid levels will have hyperlipidemia in adulthood [2].

Dyslipidemia among children shows a rapidly increasing trend which could cause epidemic of premature CVD [4]; hence, it seems very critical to identify children and adolescents with dyslipidemia, in order to design and implement lifestyle or pharmacological interventions that might be effective in preventing hyperlipidemia and atherosclerosis in adulthood [5-8].

The National Cholesterol Education program (NCEP) has defined one set of cutoff points for classifying children and adolescents, aged 2–19 years, with normal, borderline and high levels of total cholesterol (TC), low density lipoprotein cholesterol (LDL-C), high density lipoprotein cholesterol (HDL-C) and triglycerides (TG), regardless of age and sex [9]. Since lipid levels can change during maturation, and might differ in each gender the other sets of cut points have been defined according to sex and age in adolescents, aged 12–19 years, in the National Health and Nutrition Education Survey (NHANES) [10].

There is limited data regarding comparison of these two sets of cut points for prediction of adulthood dyslipidemia. Magnussen et al. demonstrated that NCEP cut points for TC, LDL-C and TG and NHANES cut points for HDL-C yielded better predictive ability for prediction of dyslipidemia in adulthood [11]. In a previous study conducted on Tehran Lipid and Glucose Study (TLGS) participants, it was shown that 16% of children, aged 3–19 years, had TC levels over 5.17mmol/L (200 mg/dL) and 17% had LDL-C levels above 3.36 mmol/L (130 mg/dL) [12]. Considering the elevated lipid levels in Iranian children and adolescents and the importance of identifying children with dyslipidemia for prevention of adult dyslipidemia and CVD, the aims of the current study were to evaluate the prevalence of borderline and high levels of lipid measures and indexes among Iranian adolescents, based on NCEP and NHANES classifications also to and compare the ability of these cut points to predict hyperlipidemia in adulthood.

## Materials and methods

### Study population

Subjects in this study were selected from among participants of the TLGS, a prospective study conducted to determine the risk factors and outcomes for non-communicable diseases [13]. To summarize, 15005 people, aged 3 years and over, residents of district-13 of Tehran, underwent a baseline examination between February 1999 and August 2001. In the current study, 1929 participants, aged 14–19 years at baseline (1999–2001), who had no missing data on lipid values and were not received lipid-lowering drugs, were included. Of these, 1032 subjects, aged at least 20 years, were re-assessed after a 6 year interval (2005 to 2008). The ethical committee of the Research Institute for Endocrine Sciences approved this study and informed written consent was obtained from all subjects.

### Clinical and laboratory measurements

Baseline information including demographic data, were collected by a trained interviewer, using a pretested questionnaire. Weight was measured, while subjects were minimally clothed without shoes, using digital scales (Seca 707, Seca Corp., Hanover, MD; range 0.1–150 kg) and recorded to the nearest 100 g. Height was measured in a standing position without shoes, using a stadiometer, with shoulders in normal alignment. Body mass index (BMI) was calculated as weight (kg) divided by square of height (m^2^). A blood sample, at baseline and follow up, was drawn between 7:00 and 9:00 AM from all study participants after 12–14 h overnight fasting. All the blood analyses were done at the TLGS research laboratory on the day of blood collection. TC was assayed using enzymatic colorimetric method with cholesterol esterase and cholesterol oxidase. HDL-C was measured after precipitation of the apolipoprotein B-containing lipoproteins with phosphotungstic acid and TGs was assayed using an enzymatic colorimetric method with glycerol phosphate oxidase. These analyses were performed using commercial kits (Pars Azmoon Inc., Tehran, Iran) and a Selectra 2 auto analyzer (Vital Scientific, Spankeren, The Netherlands). LDL-C was calculated from serum TC, TGs and HDL-C concentration, using the Friedewald formula [14]. Non-HDL-C was calculated by subtracting HDL-C from TC; TC/HDL-C and TG/HDL-C were calculated by dividing TC and TG by HDL-C respectively. The intra- and inter-assay coefficients of variation (CV) for glucose at baseline and follow- up phases were both less than 2.2%. For TC and HDL- C, intra- and inter-assay CVs were less than 2.0% and 2.8%, respectively in both phases. Intra- and inter-assay CVs were less than 1.9% for TGs in baseline and follow- up examinations.

### Classification of lipid measures

Based on NCEP and NHANES classifications, adolescents were categorized in normal, borderline and high lipid groups. To identify the cut points of non-HDL-C, TC/HDL-C and TG/HDL-C, we used the cutoffs of HDL-C, TC and TG in each of the classifications; e.g., high TG cutoff divided by low HDL-C cutoff according to NCEP, yielded high TG/HDL-C cutoff in NCEP classification. Adolescent dyslipidemia was defined as having at least one high level of lipid measures or indexes. The adolescents, who were not classified as having dyslipidemia but had at least one lipid measure or index in borderline category was defined as borderline dyslipidemia. Adults were classified using NCEP Adult Treatment Panel guidelines as having high TC if their TC levels were ≥ 6.19 mmol/L, high LDL if LDL-C levels ≥ 4.12 mmol/L, low HDL-C if HDL-C levels ≤ 1.036 mmom/L, high TG if TG levels ≥ 2.26 mmol/L, high non-HDL-C if non-HDL-C ≥ 5.15, high TC/HDL-C if TC/HDL-C levels ≥ 5.97, or high TG/HDL-C if TG/HDL-C levels were ≥ 2.18 mmom/L [15]. Nine of the subjects at follow up reported taking lipid-lowering drugs and were hence classified as having elevated adult lipid levels.

### Statistical analyses

Logistic regression analysis was used to assess the association between lipid measure categories at baseline and subsequent high lipid levels in adulthood. Odds ratios (OR) with 95% confidence interval (CI) were calculated for borderline and high categories of lipids, compared to normal levels as the reference group. Interactions between each lipid classification for prediction of developing high adult levels with age and sex were tested using the log-likelihood ratio test; there were no significant interactions between lipid cutoffs and age or sex. Multivariable ORs were adjusted for sex, baseline age and change in BMI rank between adolescence and adulthood.

Area under the receiving characteristics curve (AUC) was used to assess the predictive ability of each adolescent lipid classification and AUC, were compared using roccomp command in STATA 11(StataCorp LP, College Station, Texas).

## Results

**Figure 1:**
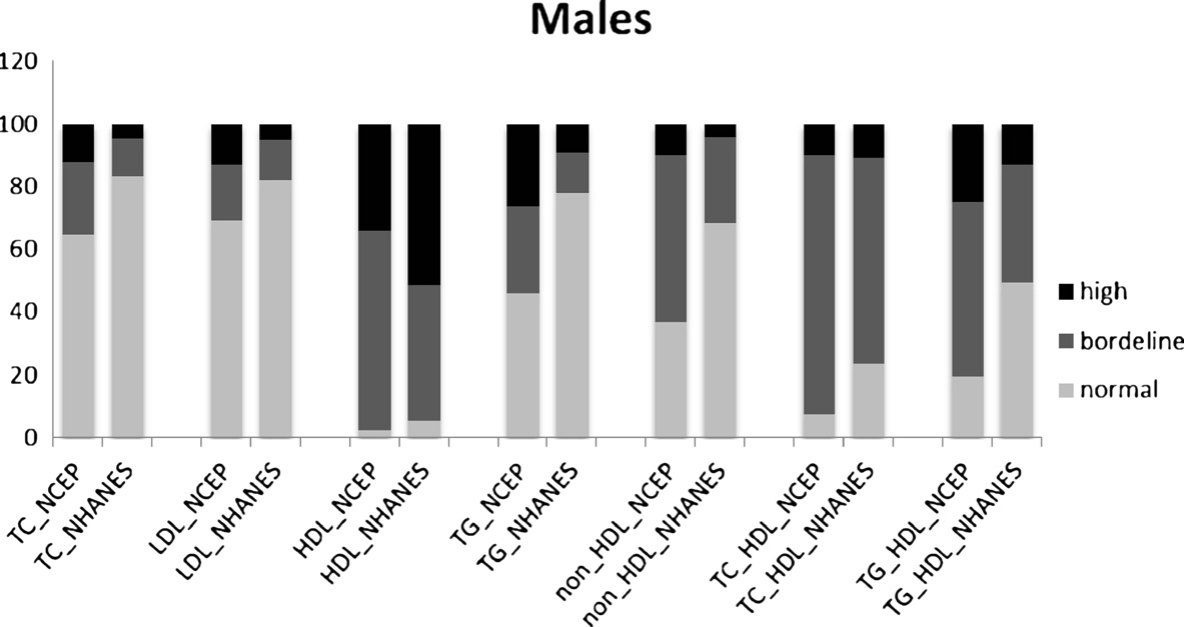
**Prevalence of normal, borderline and high lipoproteins levels in males aged 14-19 years with two sets of classifications.** NCEP: The National Cholesterol Education program; NHANES: National Health and Nutrition Education Survey; TC: total cholesterol; LDL-C: low density lipoprotein cholesterol; HDL-C: high density lipoprotein cholesterol; TG: triglycerides. *For HDL levels, the black color represented Low level of HDL, the dark grey represented borderline level and grey color represented high level of HDL-C.

**Figure 2:**
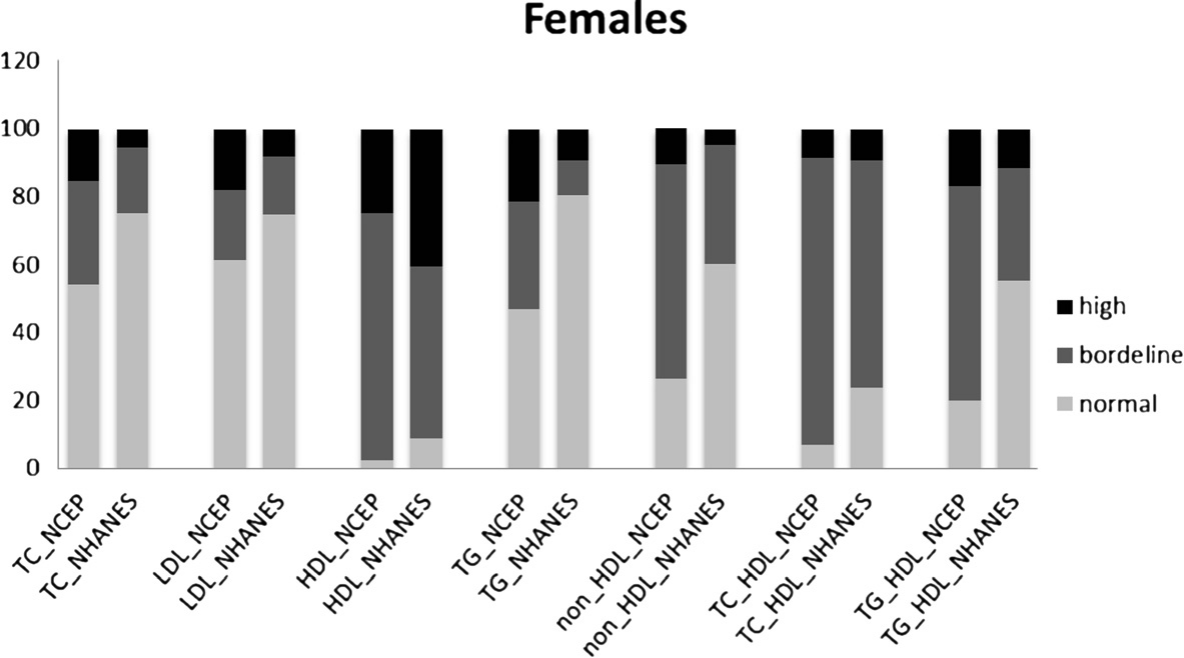
**Prevalence of normal, borderline and high lipoproteins levels in females aged 14-19 years with two sets of classifications.** NCEP: The National Cholesterol Education program; NHANES: National Health and Nutrition Education Survey; TC: total cholesterol; LDL-C: low density lipoprotein cholesterol; HDL-C: high density lipoprotein cholesterol; TG: triglycerides. *For HDL levels, the black color represented Low level of HDL, the dark grey represented borderline level and grey color represented high level of HDL-C.

The prevalences of normal, borderline and high levels of lipid measures and indexes, according to NCEP and NHANES classifications, in 1929 male and female participants, aged 14–19 years, are shown in Figures 1 and 2. The proportion of adolescents of both genders, with normal lipid measure and indexes were lower in the NCEP than in the NHANES classification. According to the NCEP classification, in males the prevalences of high TC, LDL-C, TG and low HDL-C were 12.1%, 12.9%, 26.1% and 34.2% respectively; in females the corresponding prevalences were 15.4%, 17.9%, 21.4% and 25.0%, respectively. Based on NHANES cut points, the prevalence of high TC, LDL-C, TG and low HDL-C among males were 4.4%, 5%, 9% and 51.6% and among females, these were 5.5%, 8%, 9.2% and 40.2%, respectively. Overall, low HDL-C and high TG levels tended to be more prevalent in male as compared to female adolescents in both classifications, while, vice-versa, high TC and LDL-C were higher in females than males.

As seen in Table 1, there were no significant differences in baseline characteristics between the adolescents who participated in the follow up study and those who did not.

**Table 1:** Baseline characteristics of adolescents who participated in the follow up study and those who did not

	**Participants**	**Non-participants**	**P-value**
Age (years)	16.45 ± 1.72	16.41 ± 1.69	0.6
Body mass index (kg/m^2^)	21.49 ± 4.37	21.23 ± 3.87	0.2
Total cholesterol (mmol/L)	4.26 ± 0.85	4.31 ± 0.84	0.2
LDL-C (mmol/L)	2.62 ± 0.75	2.66 ± 0.74	0.2
HDL-C (mmol/L)	1.09 ± 0.25	1.12 ± 0.26	0.05
Non-HDL-C (mmol/L)	3.17 ± 0.85	3.20 ± 0.84	0.4
Triglycerides (mmol/L)	1.22 ± 0.63	1.20 ± 0.60	0.3
TG/HDL-C (mmol/L)	1.24 ± 0.87	1.17 ± 0.74	0.08
TC/HDL-C (mmol/L)	4.08 ± 1.21	4.04 ± 1.17	0.5

LDL-C; low density cholesterol, HDL-C; high density cholesterol, TG; triglycerides, TC; total cholesterol. Mean ± standard deviation were used to expressed for variables; comparison between baseline characteristics of participants and non-participants were done by Fisher Exact test.

Table 2 presents the adjusted ORs of borderline and abnormal levels of adolescent lipid measures according to the NCEP and NHANES classifications for prediction of high lipid levels in adulthood. High categories of all lipid measures according to both NCEP and NHANES classifications were significantly associated with development of abnormal lipid levels during adulthood. The ORs of NHANES cutoffs for high levels of lipid measures and indices were higher than ORs of those based on the NECP classification, except for HDL-C, which had a very wide CI in the NCEP classification; this might be because, in the NCEP normal category of HDL-C, only one case developed low HDL-C in adulthood. The borderline categories of non-HDL-C and TC/HDL-C, according to NCEP did not show any significant association with high levels of these parameters in adulthood; whereas those who had borderline levels of non-HDL-C according to NHANES cut points had approximately a fourfold increased risk; also the borderline values of TC/HDL-C by the NHANES classification was associated with an almost 2.5 increase in risk of incident high TC/HDL-C compared with normal ones.

**Table 2:** Odds Ratios and 95% confidence interval for incident adulthood high lipids levels according to adolescent borderline and high lipid levels with NCEP and NHANES cut points

	**NCEP**		**NHANES**	
**Adolescent lipid classification**	**n/N**	**Odds Ratios (95CI)**	**n/N**	**Odds Ratios (95CI)**
Total cholesterol				
Normal	8/593	1	15/809	1
Borderline	9/300	2.3 (0.8-6.4)	12/172	13.5 (5.4-33.7)
High	19/138	5.2 (2.3-11.8)	9/50	15.2 (5.7-40.3)
LDL-C				
Normal	10/662	1	14/799	1
Borderline	8/200	2.6 (0.94-7.08)	11/157	5.2(2.2-12.4)
High	23/163	12.7 (5.6-28.9)	16/69	28.2(11.6-68.7)
Triglycerides				
Normal	24/482	1	38/826	1
Borderline	30/315	1.8(0.8-3.9)	11/113	1.8(0.8-4.1)
High	49/234	5.7(2.9-11.4)	24/92	8.4(4.3-16.4)
HDL-C				
Normal	1/29	1	266/456	1
Borderline	205/721	11.6(1.5-88.6)	114/489	1.7(0.8-3.4)
Low	185/282	58.1(7.5-450.0)	11/86	7.8(3.9-15.6)
Non-HDL-C				
Normal	4/316	1	9/654	1
Borderline	14/610	1.5(0.5-4.8)	15/333	3.69(1.5-9.0)
High	16/105	12.6(4.0-40.0)	10/44	26.3(8.9-77.0)
TC/HDL-Ca				
Normal	2/76	1	5/243	1
Borderline	42/870	1.7(0.4-7.4)	38/695	2.5(1.0-6.6)
High	29/85	16.9(3.7-77.0)	30/93	21.5(7.7-60.5)
TG/HDL-C				
Normal	8/205	1	26/550	1
Borderline	46/635	2.6(0.9-7.1)	41/366	5.2(2.2-12.4)
High	49/191	12.7(5.6-28.9)	36/115	28.2(11.6-68.7)

Odds ratios were adjusted for sex, baseline age and change in body mass index rank between adolescents and adulthood. CI: confidence interval; NCEP: The National Cholesterol Education program; NHANES: National Health and Nutrition Education Survey; TC: total cholesterol; LDL-C: low density lipoprotein cholesterol; HDL-C: high density lipoprotein cholesterol; TG: triglycerides.

**Figure 3:**
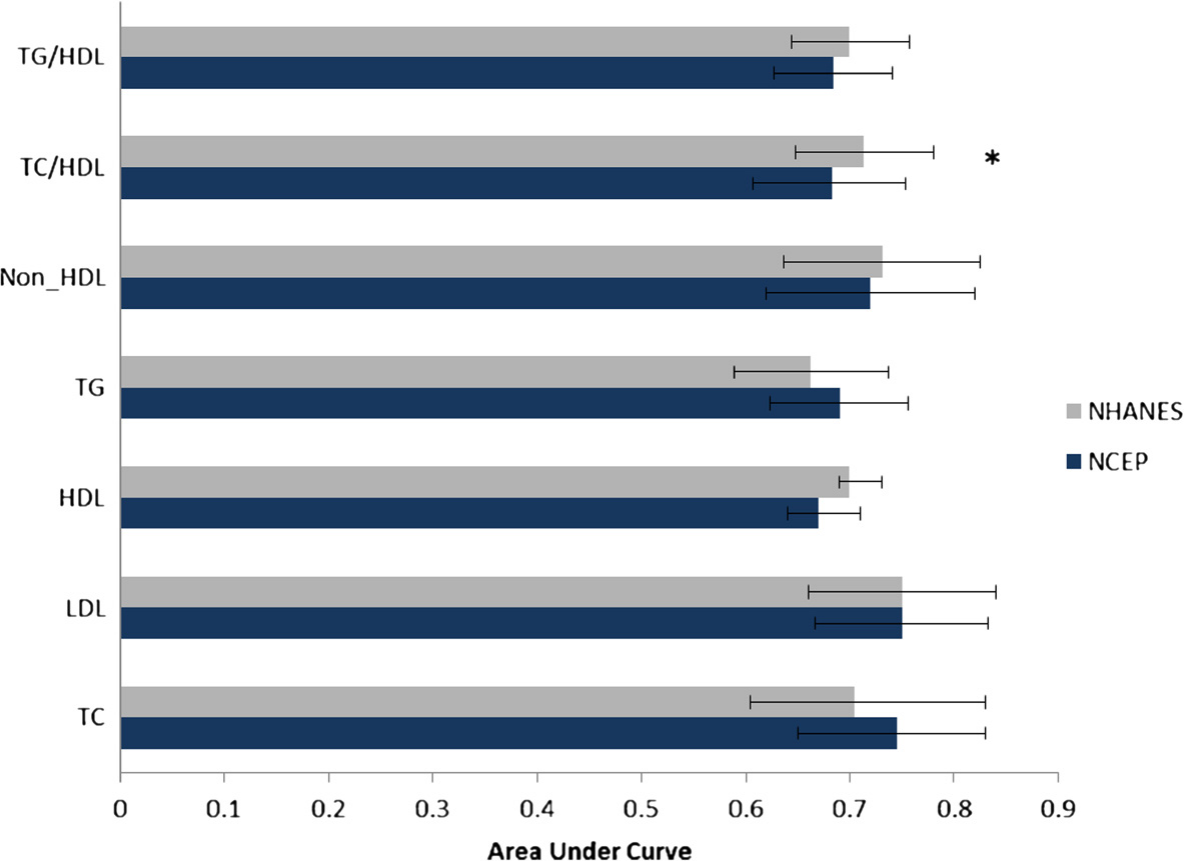
**Area under the receiving characteristics curve of each lipid measure using both classifications: NCEP: The National Cholesterol Education program; NHANES: National Health and Nutrition Education Survey; TC: total cholesterol; LDL-C: low density lipoprotein cholesterol; HDL-C: high density lipoprotein cholesterol; TG: triglycerides.** * P<0.05 for difference between two classifications.

To compare the predictive abilities of these two classifications, we used AUC (Figure 3). Almost all the NCEP and NHANES cut points had similar predictive abilities except for TC/HDL-C, indicating that NHANES classification had better power for prediction of high adulthood TC/HDL-C than NCEP cut points (≈ 71% vs. 68%).

## Discussion

In this community based study that assessed lipid levels in children by both NCEP and NHANES lipid classifications, findings showed a high prevalence of abnormal lipid levels in Iranian adolescents aged 14–19 years. We found that both the dyslipidemia cut points were able to predict high lipid levels after 6 years of follow up, independent of obesity; the risk of developing high lipoprotein levels in young adulthood was significantly higher in adolescents who were in the borderline- and high categories of lipids compared with those in the normal category, a risk which increased moving from borderline to high lipid levels. The predictive powers of NCEP and NHANES classifications for all lipid measures were similar except for TC/HDL-C values, in which NHANES cut points had higher discriminatory power (as assessed by AUC) than NCEP.

Previous studies in Iran, conducted on children and adolescents aged 3–19 years, showed that Tehranian children had higher TC, TGs and LDL-C levels and lower HDL-C levels than Americans and Greeks [12]. In this study for the first time we reported the prevalence of borderline and high lipid levels in Iranian adolescents, according to NCEP and NHANES cut points. The prevalence of borderline and high levels of TC, LDL-C and TGs were higher with NCEP classification, compared to NHANES, however borderline and low level of HDL-C was higher with NHANES classification. The overall prevalence of high and borderline TC and LDL-C levels observed in the current study were similar to those of the Bogalusa study [11] in both genders. The prevalences of high TC and LDL-C, in the Young Finns studies [11] were higher than those in ours. Compared to both the Bogalusa and Young Finns studies [11], the prevalence of high and borderline TG levels and low HDL-C were higher in the current study, especially in females.

This high prevalence of abnormal TG, especially HDL-C levels, in our population might be due to dietary patterns and low physical activity levels [16-18]. The usual Iranian diet is wheat-based and during 1985 and 1995, urban households increased their bread consumption by almost 25% [17], indicating that hypertriglyceridemia in our population may be induced by high carbohydrate diets, leading to the production and secretion of TG and very low density lipoprotein due to lipogenesis in the liver [19,20].

Since the prevalence of normal HDL-C, compared to high TC, was very low in our adolescents, we examined the prevalence of lipid ratios, according to different cut points, as expected a large number of adolescents had borderline levels of lipid ratios, especially TC/HDL-C. For the first time we evaluated the ability of different cut points of lipid ratios to predict high lipid levels during adulthood and showed that high values of these ratios strongly predicted high levels of lipid ratios in adulthood which are independent predictors of diabetes, hypertension and CVD in our population [21-23].

Magnussen et al [11] for the first time compared NCEP and NHANES lipoprotein classifications for development of elevated high lipid concentration in three adult cohorts. They showed that NCEP cut points for TC, LDL-C and TG and NHANES cut points for HDL-C yielded the better classification for predicting adulthood dyslipidemia [11]. Their baseline data, collected in the 1980s, were similar to NCEP data but the NHANES data were related to 1988–2002; it could be hence possible that the preference to use NCEP cut points in their study was related to distribution of lipoproteins at that time. Our baseline examinations were done between 1999 and 2001, which was almost similar to the NHANES study. In the current study there were no significant differences between predictive powers of NCEP and NHANES cut points of TC, LDL-C, HDL-C, TG, non-HDL-C and TG/HDL-C for prediction of high lipid levels in early adulthood, after almost 6 years follow up; but the TC/HDL-C cut points derived from NHANES cut offs of TC and HDL-C had higher discriminatory power than those of the NCEP classification. It still seems debatable that precisely which of these classifications are suitable for clinical settings; more population based studies are needed to clarify this issue.

In a previous study in Iran, although it was shown that there was a significant decrease in TC during puberty in males [12], the results of this study demonstrated that for prediction of young adulthood dyslipidemia, use of the NHANES classification, which considered age in setting cut points, had not superiority over the NCEP classification.

Although in general, both of the classifications showed similar predictability for adulthood dyslipidemia, NCEP seems to be more practical in clinical settings, because of single sets of cut points.

There are some limitations regarding the interpretation of our data. First, lipid values were based on one measurement at baseline and follow up, thus creating a potential bias about the accurate classification of lipids and regression dilution, especially for TG. The second limitation was the short duration of follow up and the limited number of subjects. Third, Lipids related gene expression have an effect on the dyslipidemia but there was no data regarding to genetic characteristics in the TLGS; although genetic determines cholesterol metabolism partly, but this effect can be interfered with environmental factors. Accordingly, some studies confirm the importance of lifestyle changes from adolescence to minimize the risk of future CVD [24]. Finally, there was no dietary data in adolescent population in TLGS; although, we have adjusted BMI rank in multivariable model which can be an indicator of nutritional status.

In this study for the first time we presented the prevalence of normal, borderline and high lipid measures in Iranian adolescents, according to universal lipid classifications and also evaluated different cut points of lipid ratios for prediction of high lipid ratios in young adulthood.

In conclusion, this study indicates that there were no significant differences between NCEP and NHANES classifications for prediction of high lipoprotein level in adulthood except for TC/HDL-C. The NHANES cut points for TC/HDL-C yielded higher predictive ability than NCEP. We can use NCEP cut points can be used in clinical setting because of their simple application.

## Competing interests

The authors declare that they have no competing interests.

## Authors’ contributions

MH participated in statistical analysis and interpretation of data and drafting the manuscript .MT participated in the conception and design of the study; interpretation of data and drafting the manuscript. RM revised the manuscript for important intellectual content. DK participated in statistical analyses. FA participated in its design and coordination. FH participated in the conception and design of the study and revised the manuscript for important intellectual content and final approval .All authors read and approved the final manuscript.
